# Supplementary material for: Iterative improvement in the automatic modular design of robot swarms
Source: PeerJ Comput Sci. 2020 Dec 7;6:e322. doi: 10.7717/peerj-cs.322 (PMC7924708; doi:10.7717/peerj-cs.322)
Supplement: Supplemental Information 3 [file peerj-cs-06-322-s003.zip › argos3/doc/api/standalone/a00606_source.html]

ARGoS: plugins/simulator/physics\_engines/dynamics2d/dynamics2d\_engine.cpp Source File


- Main Page
- Related Pages
- Namespaces
- Classes
- Files

- File List
- File Members

# plugins/simulator/physics\_engines/dynamics2d/dynamics2d\_engine.cpp

Go to the documentation of this file.

```
00001 
00007 #include "dynamics2d_engine.h"
00008 #include "dynamics2d_model.h"
00009 #include "dynamics2d_gripping.h"
00010 
00011 #include <argos3/core/simulator/simulator.h>
00012 #include <argos3/core/simulator/entity/embodied_entity.h>
00013 
00014 #include <cmath>
00015 
00016 namespace argos {
00017 
00018    /****************************************/
00019    /****************************************/
00020 
00021    CDynamics2DEngine::CDynamics2DEngine() :
00022       m_fStaticHashCellSize(0.1f),
00023       m_fActiveHashCellSize(2.0f * 0.085036758f),
00024       m_nStaticHashCells(1000),
00025       m_nActiveHashCells(1000),
00026       m_ptSpace(NULL),
00027       m_ptGroundBody(NULL),
00028       m_fElevation(0.0f) {
00029    }
00030 
00031    /****************************************/
00032    /****************************************/
00033 
00034    void CDynamics2DEngine::Init(TConfigurationNode& t_tree) {
00035       try {
00036          /* Init parent */
00037          CPhysicsEngine::Init(t_tree);
00038          /* Parse XML */
00039          GetNodeAttributeOrDefault(t_tree, "static_cell_size", m_fStaticHashCellSize, m_fStaticHashCellSize);
00040          GetNodeAttributeOrDefault(t_tree, "active_cell_size", m_fActiveHashCellSize, m_fActiveHashCellSize);
00041          GetNodeAttributeOrDefault(t_tree, "static_cells",     m_nStaticHashCells,    m_nStaticHashCells);
00042          GetNodeAttributeOrDefault(t_tree, "active_cells",     m_nActiveHashCells,    m_nActiveHashCells);
00043          GetNodeAttributeOrDefault(t_tree, "elevation",        m_fElevation,          m_fElevation);
00044          /* Override volume top and bottom with the value of m_fElevation */
00045          if(!GetVolume().TopFace)    GetVolume().TopFace    = new SHorizontalFace;
00046          if(!GetVolume().BottomFace) GetVolume().BottomFace = new SHorizontalFace;
00047          GetVolume().TopFace->Height    = m_fElevation;
00048          GetVolume().BottomFace->Height = m_fElevation;
00049          /* Initialize physics */
00050          cpInitChipmunk();
00051          cpResetShapeIdCounter();
00052          /* Used to attach static geometries so that they won't move and to simulate friction */
00053          m_ptGroundBody = cpBodyNew(INFINITY, INFINITY);
00054          /* Create the space to contain the movable objects */
00055          m_ptSpace = cpSpaceNew();
00056          /* Subiterations to solve constraints.
00057             The more, the better for precision but the worse for speed
00058          */
00059          m_ptSpace->iterations = GetIterations();
00060          /* Resize the space hash.
00061             This has dramatic effects on performance.
00062             TODO: - find optimal parameters automatically (average entity size)
00063             cpSpaceReindexStaticHash(m_ptSpace, m_fStaticHashCellSize, m_nStaticHashCells);
00064             cpSpaceResizeActiveHash(m_ptSpace, m_fActiveHashCellSize, m_nActiveHashCells);
00065          */
00066          /* Gripper-Gripped callback functions */
00067          cpSpaceAddCollisionHandler(
00068             m_ptSpace,
00069             SHAPE_GRIPPER,
00070             SHAPE_GRIPPABLE,
00071             BeginCollisionBetweenGripperAndGrippable,
00072             ManageCollisionBetweenGripperAndGrippable,
00073             NULL,
00074             NULL,
00075             NULL);
00076       }
00077       catch(CARGoSException& ex) {
00078          THROW_ARGOSEXCEPTION_NESTED("Error initializing the dynamics 2D engine \"" << GetId() << "\"", ex);
00079       }
00080    }
00081 
00082    /****************************************/
00083    /****************************************/
00084 
00085    void CDynamics2DEngine::Reset() {
00086       for(CDynamics2DModel::TMap::iterator it = m_tPhysicsModels.begin();
00087           it != m_tPhysicsModels.end(); ++it) {
00088          it->second->Reset();
00089       }
00090       cpSpaceReindexStatic(m_ptSpace);
00091    }
00092 
00093    /****************************************/
00094    /****************************************/
00095 
00096    void CDynamics2DEngine::Update() {
00097       /* Update the physics state from the entities */
00098       for(CDynamics2DModel::TMap::iterator it = m_tPhysicsModels.begin();
00099           it != m_tPhysicsModels.end(); ++it) {
00100          it->second->UpdateFromEntityStatus();
00101       }
00102       /* Perform the step */
00103       for(size_t i = 0; i < GetIterations(); ++i) {
00104          cpSpaceStep(m_ptSpace, GetPhysicsClockTick());
00105       }
00106       /* Update the simulated space */
00107       for(CDynamics2DModel::TMap::iterator it = m_tPhysicsModels.begin();
00108           it != m_tPhysicsModels.end(); ++it) {
00109          it->second->UpdateEntityStatus();
00110       }
00111    }
00112 
00113    /****************************************/
00114    /****************************************/
00115 
00116    void CDynamics2DEngine::Destroy() {
00117       /* Empty the physics model map */
00118       for(CDynamics2DModel::TMap::iterator it = m_tPhysicsModels.begin();
00119           it != m_tPhysicsModels.end(); ++it) {
00120          delete it->second;
00121       }
00122       m_tPhysicsModels.clear();
00123       /* Get rid of the physics space */
00124       cpSpaceFree(m_ptSpace);
00125       cpBodyFree(m_ptGroundBody);
00126    }
00127 
00128    /****************************************/
00129    /****************************************/
00130 
00131    size_t CDynamics2DEngine::GetNumPhysicsModels() {
00132       return m_tPhysicsModels.size();
00133    }
00134 
00135    /****************************************/
00136    /****************************************/
00137 
00138    bool CDynamics2DEngine::AddEntity(CEntity& c_entity) {
00139       SOperationOutcome cOutcome =
00140          CallEntityOperation<CDynamics2DOperationAddEntity, CDynamics2DEngine, SOperationOutcome>
00141          (*this, c_entity);
00142       cpResetShapeIdCounter();
00143       return cOutcome.Value;
00144    }
00145 
00146    /****************************************/
00147    /****************************************/
00148 
00149    bool CDynamics2DEngine::RemoveEntity(CEntity& c_entity) {
00150       SOperationOutcome cOutcome =
00151          CallEntityOperation<CDynamics2DOperationRemoveEntity, CDynamics2DEngine, SOperationOutcome>
00152          (*this, c_entity);
00153       return cOutcome.Value;
00154    }
00155 
00156    /****************************************/
00157    /****************************************/
00158 
00159    struct SDynamics2DSegmentHitData {
00160       TEmbodiedEntityIntersectionData& Intersections;
00161       const CRay3& Ray;
00162 
00163       SDynamics2DSegmentHitData(TEmbodiedEntityIntersectionData& t_data,
00164                                 const CRay3& c_ray) :
00165          Intersections(t_data),
00166          Ray(c_ray) {}
00167    };
00168 
00169    static void Dynamics2DSegmentQueryFunc(cpShape* pt_shape, cpFloat f_t, cpVect, void* pt_data) {
00170       /* Get the data associated to this query */
00171       SDynamics2DSegmentHitData& sData = *reinterpret_cast<SDynamics2DSegmentHitData*>(pt_data);
00172       /* Hit found, is it within the limits? */
00173       CDynamics2DModel& cModel = *reinterpret_cast<CDynamics2DModel*>(pt_shape->body->data);
00174       CVector3 cIntersectionPoint;
00175       sData.Ray.GetPoint(cIntersectionPoint, f_t);
00176       if((cIntersectionPoint.GetZ() >= cModel.GetBoundingBox().MinCorner.GetZ()) &&
00177          (cIntersectionPoint.GetZ() <= cModel.GetBoundingBox().MaxCorner.GetZ()) ) {
00178          /* Yes, a real hit */
00179          sData.Intersections.push_back(
00180             SEmbodiedEntityIntersectionItem(
00181                &cModel.GetEmbodiedEntity(),
00182                f_t));
00183       }
00184    }
00185    
00186    void CDynamics2DEngine::CheckIntersectionWithRay(TEmbodiedEntityIntersectionData& t_data,
00187                                                     const CRay3& c_ray) const {
00188       /* Query all hits along the ray */
00189       SDynamics2DSegmentHitData sHitData(t_data, c_ray);
00190       cpSpaceSegmentQuery(
00191          m_ptSpace,
00192          cpv(c_ray.GetStart().GetX(), c_ray.GetStart().GetY()),
00193          cpv(c_ray.GetEnd().GetX()  , c_ray.GetEnd().GetY()  ),
00194          CP_ALL_LAYERS,
00195          CP_NO_GROUP,
00196          Dynamics2DSegmentQueryFunc,
00197          &sHitData);
00198    }
00199 
00200    /****************************************/
00201    /****************************************/
00202 
00203    void CDynamics2DEngine::PositionPhysicsToSpace(CVector3& c_new_pos,
00204                                                   const CVector3& c_original_pos,
00205                                                   const cpBody* pt_body) {
00206       c_new_pos.SetX(pt_body->p.x);
00207       c_new_pos.SetY(pt_body->p.y);
00208       c_new_pos.SetZ(c_original_pos.GetZ());
00209    }
00210 
00211    /****************************************/
00212    /****************************************/
00213 
00214    void CDynamics2DEngine::OrientationPhysicsToSpace(CQuaternion& c_new_orient,
00215                                                      cpBody* pt_body) {
00216       c_new_orient.FromAngleAxis(CRadians(pt_body->a), CVector3::Z);
00217    }
00218 
00219    /****************************************/
00220    /****************************************/
00221 
00222    void CDynamics2DEngine::AddPhysicsModel(const std::string& str_id,
00223                                             CDynamics2DModel& c_model) {
00224       m_tPhysicsModels[str_id] = &c_model;
00225    }
00226 
00227    /****************************************/
00228    /****************************************/
00229 
00230    void CDynamics2DEngine::RemovePhysicsModel(const std::string& str_id) {
00231       CDynamics2DModel::TMap::iterator it = m_tPhysicsModels.find(str_id);
00232       if(it != m_tPhysicsModels.end()) {
00233          delete it->second;
00234          m_tPhysicsModels.erase(it);
00235       }
00236       else {
00237          THROW_ARGOSEXCEPTION("Dynamics2D model id \"" << str_id << "\" not found in dynamics 2D engine \"" << GetId() << "\"");
00238       }
00239    }
00240 
00241    /****************************************/
00242    /****************************************/
00243 
00244    REGISTER_PHYSICS_ENGINE(CDynamics2DEngine,
00245                            "dynamics2d",
00246                            "Carlo Pinciroli [ilpincy@gmail.com]",
00247                            "1.0",
00248                            "A 2D dynamics physics engine.",
00249                            "This physics engine is a 2D dynamics engine based on the Chipmunk library\n"
00250                            "(http://code.google.com/p/chipmunk-physics).\n\n"
00251                            "REQUIRED XML CONFIGURATION\n\n"
00252                            "  <physics_engines>\n"
00253                            "    ...\n"
00254                            "    <dynamics2d id=\"dyn2d\" />\n"
00255                            "    ...\n"
00256                            "  </physics_engines>\n\n"
00257                            "The 'id' attribute is necessary and must be unique among the physics engines.\n"
00258                            "It is used in the subsequent section <arena_physics> to assign entities to\n"
00259                            "physics engines. If two engines share the same id, initialization aborts.\n\n"
00260                            "OPTIONAL XML CONFIGURATION\n\n"
00261                            "The plane of the physics engine can be translated on the Z axis, to simulate\n"
00262                            "for example hovering objects, such as flying robots. To translate the plane\n"
00263                            "2m up the Z axis, use the 'elevation' attribute as follows:\n\n"
00264                            "  <physics_engines>\n"
00265                            "    ...\n"
00266                            "    <dynamics2d id=\"dyn2d\"\n"
00267                            "                elevation=\"2.0\" />\n"
00268                            "    ...\n"
00269                            "  </physics_engines>\n\n"
00270                            "When not specified, the elevation is zero, which means that the plane\n"
00271                            "corresponds to the XY plane.\n",
00272                            "Under development"
00273       );
00274 
00275 }
```

---

Generated on 10 Jul 2018 for ARGoS by 
 1.6.1
